# Temperature response of Rubisco kinetics in *Arabidopsis thaliana*: thermal breakpoints and implications for reaction mechanisms

**DOI:** 10.1093/jxb/ery355

**Published:** 2018-11-07

**Authors:** Ryan A Boyd, Amanda P Cavanagh, David S Kubien, Asaph B Cousins

**Affiliations:** 1 School of Biological Sciences, Molecular Plant Sciences, Washington State University, Pullman, WA, USA; 2 Department of Biology, University of New Brunswick, Fredericton, NB, Canada

**Keywords:** Arabidopsis, kinetic breakpoints, membrane inlet mass spectrometery, reaction mechanisms, Rubisco, temperature

## Abstract

Enhancement of Rubisco kinetics could improve photosynthetic efficiency, ultimately resulting in increased crop yield. However, imprecise knowledge of the reaction mechanism and the individual rate constants limits our ability to optimize the enzyme. Membrane inlet mass spectrometry (MIMS) may offer benefits over traditional methods for determining individual rate constants of the Rubisco reaction mechanism, as it can directly monitor concentration changes in CO_2_, O_2_, and their isotopologs during assays. However, a direct comparison of MIMS with the traditional radiolabel method of determining Rubisco kinetic parameters has not been made. Here, the temperature responses of Rubisco kinetic parameters from *Arabidopsis thaliana* were measured using radiolabel and MIMS methods. The two methods provided comparable parameters above 25 °C, but temperature responses deviated at low temperature as MIMS-derived catalytic rates of carboxylation, oxygenation, and CO_2_/O_2_ specificity showed thermal breakpoints. Here, we discuss the variability and uncertainty surrounding breakpoints in the Rubisco temperature response and the relevance of individual rate constants of the reaction mechanisms to potential breakpoints.

## Teaser

Rubisco kinetic parameters from *Arabidopsis thaliana* were measured using radiolabel and membrane inlet mass spectrometry to determine how the reaction mechanisms and the individual rate constants change with temperature.

## Introduction

The enzyme Rubisco catalyzes the reaction of CO_2_ or O_2_ with ribulose-1,5-bisphosphate (RuBP) initiating the photosynthetic carbon reduction cycle or photorespiratory cycle, respectively (Bowes *et al.*, 1971; Andrews *et al.*, 1973). Kinetic studies on Rubisco typically report the Michaelis–Menten constants for carboxylation (*K*_C_) and oxygenation (*K*_O_), the catalytic rate of carboxylation (*k*_catCO2_) and oxygenation (*k*_catO2_), and the specificity of the enzyme for CO_2_ over O_2_ (*S*_C/O_) as these parameters are used for modeling leaf gas exchange (von Caemmerer, 2000). Each of the above Michaelis–Menten parameters is a combination of elementary rate constants that describe the reaction mechanism; however, the rate constants are less well studied as the nature of the chemical mechanism and their intermediates are uncertain (Tcherkez, 2013, 2016). Optimization of Rubisco kinetics for enhanced CO_2_ reduction has been proposed (Spreitzer and Salvucci, 2002), but this effort is limited by our current understanding of the reaction mechanism (Tcherkez *et al.*, 2006; Tcherkez, 2013).

**Fig. 1.**
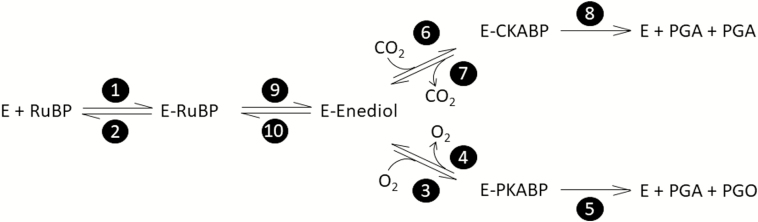
Elementary reactions of Rubisco-catalyzed carboxylation and oxygenation (Farquhar,1979). Each reaction, forward and reverse, is numbered in a filled circle following the numbering from Farquhar (1979). Steps 8 and 5 are written as irreversible reactions. Step 8 includes cleavage, hydration, and reprotonation as a single step. Step 5 includes cleavage and hydration as a single step. Each step is associated with a rate constant (*k*) and energy of activation (Δ*G*^‡^) following the same numbering as shown in the filled circles. Abbreviations are as follows: E, free activated enzyme; RuBP, d-ribulose-1,5-bisphosphate; E-RuBP; enzyme-bound RuBP; E-Enediol, enzyme-bound 2,3-enediolate form of RuBP; CO_2_, carbon dioxide; E-CKABP, enzyme-bound carboxyketone intermediate; PGA, 3-phospho-d-glycerate; O_2_, oxygen; E-PKABP, peroxo intermediate; PGO, 2-phosphoglycolate.

The carboxylation and oxygenation reaction mechanisms can be separated into elementary rate constant as originally proposed by Farquhar (1979), reviewed by Tcherkez (2013), and reproduced in Fig. 1. Since the initial description of the reaction mechanism (Hurwitz *et al.*, 1956), there has been slow progress in defining rate constants due to experimental difficulties in isolating their individual effects. However, the use of membrane inlet mass spectrometry (MIMS) to study Rubisco kinetics may hold promise. The traditional radiolabel method used in most Rubisco publications relies on ^14^C assays to determine *k*_catCO2_, *K*_C_, and *K*_O_, a separate ^3^H assay to determine *S*_C/O_, leaving *k*_catO2_ to be calculated. Alternatively, the MIMS assay simultaneously measures changing concentrations of CO_2_ and O_2_, and can therefore determine all kinetic parameters with a single assay (Cousins *et al.*, 2010; Boyd *et al.*, 2015). An advantage of the MIMS method is that in addition to the abundant isotopologs of CO_2_ (^12^CO_2_) and O_2_ (^16^O_2_), the system can monitor less abundant stable isotopologs such as ^13^CO_2_ and ^16^O^18^O. Measurements of primary kinetic isotope effects have been useful in defining enzyme reaction mechanisms (O’Leary *et al.*, 1992); therefore, the MIMS system may provide new information regarding the individual rate constants. At 25 °C the MIMS method has been used for determining both Rubisco carbon fractionation (McNevin *et al.*, 2006, 2007; Tcherkez *et al.*, 2013), and Michaelis–Menten constants of the carboxylation (*v*_c_) and oxygenation (*v*_o_) reactions (Cousins *et al.*, 2010). Additionally, it was used to determine the temperature dependencies of the Rubisco kinetic parameters in the C_4_ species *Setaria viridis*, where the Arrhenius energy of activation (*E*_a_) is used to describe the temperature dependence of chemical reaction rates (Boyd *et al.*, 2015). However, previous work using the radiolabel method suggests lower *E*_a_ values for *V*_cmax_ in C_4_ species than that measured by Boyd *et al.* (2015) (Sage, 2002; Kubien *et al.*, 2003; Perdomo *et al.*, 2015; Sharwood *et al.*, 2016). This suggests that comparisons between the MIMS *E*_a_ values and the traditional radiolabel method are needed.

Here we measured the temperature response of Rubisco kinetic parameters from *Arabidopsis thaliana* using two methods. First, we used the traditional method involving the use of radiolabeled substrate and analysis of labeled products following the reaction in known concentrations of CO_2_ and O_2_ (Jordan and Ogren, 1981). Secondly, we used the MIMS method following the simultaneous consumption of CO_2_ and O_2_ over time, giving a direct measure of *v*_c_, *v*_o_, CO_2_, and O_2_, leading to simultaneous determination of *k*_catCO2_, *k*_catO2_, *K*_C_, *K*_O_, and *S*_C/O_ (Cousins *et al.*, 2010; Boyd *et al.*, 2015). Additionally, for the radiolabel method, we compared curve fitting CO_2_ responses to determine *K*_C_ and *k*_catCO2_ simultaniously in an O_2_-free buffer, and *k*_catCO2_ determined at a single bicarbonate concentration in open air. The latter is a common practice for determining *k*_catCO2_ temperature responses (Tieszen and Sigurdson, 1973; Sage *et al.*, 1995; Crafts-Brander and Salvucci, 2000; Pittermann and Sage, 2000; Sage, 2002; Kubien *et al.*, 2003; Perdomo *et al.*, 2015).

Recently, the existence of thermal breakpoints in the *k*_catCO2_ temperature response was highlighted as a source of variability in the Rubisco temperature response literature (Sharwood *et al.*, 2016). Thermal breakpoints occur when *E*_a_ values differ between temperature ranges. Initial observations of breakpoints in *V*_cmax_ temperature responses were determined to be a methodological artifact due to the use of a single bicarbonate concentration at all temperatures and were corrected by varying the bicarbonate concentration with temperature (Björkman and Pearcy, 1970). However, breakpoints were later observed for *k*_catCO2_, *k*_catO2_, and *K*_C_ at 15 °C using a curve fitting method (Badger and Collatz, 1977). It was suggested that these breakpoints could be due to changes in rate-limiting steps of the reaction mechanism caused by changes in enzyme conformation (Badger and Collatz, 1977). An additional breakpoint was reported in the *k*_catCO2_ of *Oryza sativa* at 22 °C (Sage, 2002), and Kubien *et al.* (2003) observed different temperature responses when *k*_catCO2_ was measured from 0 °C to 12 °C compared with 18 °C to 42 °C in *Flaveria bidentis*. Most recently, Sharwood *et al.* (2016) observed breakpoints in *k*_catCO2_ at 25 °C for Panicoid grasses when using a curve fitting method. Inconsistencies are evident between studies, and it is unclear if breakpoints are universal to all temperature response studies of plant Rubisco. Here, we discuss the possible causes of breakpoints, focusing on the three previously proposed causes of breakpoints: erroneous bicarbonate concentrations, changes in the rate-limiting step of the reaction mechanism, and deactivation of Rubisco at low temperature, using the radiolabel and MIMS data sets reported here.

## Materials and methods

### Plant growth

Plants for the radiolabel method were grown and assayed at the University of New Brunswick, Fredericton, Canada. *Arabidopsis thaliana* (Col-0) seeds were stratified for 3 d at 4 °C on Promix (Plant Products, Brampton, Canada), transferred to a growth chamber (E-15, Conviron, Winnipeg, Manitoba, Canada), and grown under a photoperiod of 10 h light and14 h dark, day/night temperatures of 20/18 °C, and a photosynthetic photon flux density (PPFD) of 300 μmol m^−2^ s^−1^. Plants were watered with modified Hoagland’s solution as needed.

Plants for MIMS were grown and assayed at Washington State University, Pullman, Washington, USA. Seeds of *A. thaliana*, ecotype Col-0, were placed in 2 liter pots containing commercial soil (LC1 Sunshine Mix, Sun Gro Horticulture, Vancouver, Canada) and grown in an environmental growth chamber (Biochambers GC-16, Winnipeg, Manitoba, Canada) at a PPFD of 300 µmol m^−2^ s^−1^ at plant height, relative humidity was not controlled, and day/night temperature was 23/18 °C, with a 14 h photoperiod and 10 h of dark. Plants were fertilized weekly (Peters 20-20- 20, Allentown, PA, USA) and watered as needed.

### Sampling for radiolabel analysis

Leaf punches were obtained at mid-day, flash-frozen in liquid nitrogen, and stored at –80 °C until extraction. Leaf tissue was ground (1.1 cm^2^ disks, ~20 mg) in a Tenbroeck glass tissue homogenizer containing 3 ml of ice-cold extraction buffer [100 mM HEPES pH 7.6, 2 mM Na-EDTA, 5 mM MgCl_2_, 5 mM DTT, 10 mg ml^−1^ polyvinylpolypyrolidone (PVPP), 2% (v/v) Tween-80, 2 mM NaH_2_PO_4_, 12 mM amino-*n*-capronic acid, and 2 mM benzamidine] and 50 μl of Protease inhibitor cocktail (Sigma, St. Louis, MO, USA). This leaf homogenate was centrifuged at 16 000 *g* at 4 °C for 60 s. The resulting supernatant was then desalted using an Econo Pac 1-DG column (Bio-Rad), and aliquots were incubated with 20 mM MgCl_2_ and 10 mM NaHCO_3_ at 30 °C for 20 min to carbamylate Rubisco fully. Rubisco content (number of active sites) was quantified using the [^14^C]carboxy-arabinitol bisphosphate (^14^CABP)-binding assay (Ruuska *et al.*, 1998; Kubien *et al.*, 2011).

### Sampling for MIMS analysis

The youngest fully expanded leaves of plants 30–40 d after planting were sampled for Rubisco extraction. The mid vein was removed and ~2 g of leaf tissue was ground in 2 ml of ice-cold extraction buffer [100 mM HEPES pH 7.8, 10 mM DTT, 25 mM MgCl_2_, 1 mM EDTA, 10 mM NaHCO_3_, 1% (g ml^–1^) PVPP, 0.5% (v/v) Triton X-100] with a mortar and pestle on ice. Protease inhibitor cocktail (P9599, Sigma-Aldrich), 67 µl per 2 g of fresh leaf tissue, was added to the extraction buffer before grinding. The homogenized extract was centrifuged at 4 °C, for 10 min, at 17 000 *g*. The supernatant was collected and desalted using an Econo Pac 10DG column (Bio-Rad), filtered through a Millex GP 33 mm syringe-driven filter unit (Millipore), and then centrifuged using Amicon Ultra Ultracel 30K centrifugal filters (Millipore) at 4 °C for 1 h at 3000 *g*. The layer maintained above the filter unit was collected, brought to 20% glycerol (v/v), flash-frozen in liquid nitrogen, and stored at –80 °C until measured. Rubisco content was determined as described above.

### Radiolabel measurement of Rubisco kinetic parameters

The maximum carboxylation rate of fully activated Rubisco (*V*_cmax_) was measured following the methods of Kubien *et al.* (2011) from 0 °C to 35 °C, by the incorporation of ^14^C into acid-stable products. This method is later referred to as the ‘single point’ method. Assays were initiated by the addition of 50 μl of activated extract (as described above) to 250 μl of assay buffer [100 mM Bicine-NaOH (pH 8.2), 1 mM Na-EDTA, 20 mM MgCl_2_, 5 mM DTT, 400 μM RuBP, and 11 mM NaH^14^CO_3_ (~700 Bq nmol^−1^)] and stopped after 30–60 s by adding 250 μl of 1 M formic acid. Samples were dried at 90 °C, and ^14^C acid-stable products were counted using a scintillation counter (LS-6500, Beckman-Coulter). The catalytic rate of carboxylation (*k*_catCO2_) was calculated using the equation

$$k_{\text{catCO2}}=\frac{V_{\text{cmax}}}{\text{active sites}}$$ (1)

where active sites are measured by the ^14^CABP method described above. It was assumed that there is a one to one relationship between the moles of ^14^CABP and active sites, resulting in units for *k*_catCO2_ of mol CO_2_ mol^−1^ active site s^−1^ that simplifies to s^−1^.

Michaelis–Menten parameters for CO_2_ (*K*_C_), and apparent *K*_*C*_ at 21% O_2_ [*K*_C (21% O2)_] were determined by assaying the initial rate of Rubisco carboxylation (*v*_c_) in 7 ml septum-sealed, N_2_-sparged vials over a range of seven NaH^14^CO_3_ concentrations (Paul *et al.*, 1991; Kubien *et al.*, 2008). Concentrations of NaHCO_3_ varied depending on temperature (e.g. 0.01–3.0 mM at 10 °C, versus 0.3–13.0 mM at 35 °C). The temperature effect on pH using p*K*_a_ values (Edsall and Wyman, 1958) to calculate the CO_2_ concentration was incorporated and the Henry coefficients (Sander, 2015) were used to account for the temperature effect on CO_2_ solubility (see Supplementary Table S1 at *JXB* online). Assays were initiated by injecting 50 μl of the activated extract into vials containing CO_2_-free assay buffer [100 mM Bicine-NaOH (pH 8.2 at 25 °C), 20 mM MgCl_2_, 1 mM Na_2_-EDTA, 400 μM RuBP, and 10 μg ml^−1^ carbonic anhydrase], stopped after 30–60 s by adding 250 μl of 1 M formic acid, and counted as described above. The response of *v*_c_ to partial pressures of CO_2_ were fit to the Michaelis–Menten equation

$$v_{\text{c}}=\frac{V_{\text{cmax}}\;CO_{\text{2}}}{CO_{\text{2}}+K_{\text{C}}}$$ (2)

in SigmaPlot (Systat Software, San Jose, CA, USA) solving for *V*_cmax_ and *K*_C_. This analysis, referred to as the ‘curve fitting’ method, gave a separate temperature response of *k*_catCO2_ from the single point method described above. From the same extract, the apparent *K*_*C*_ at 21% O_2_ [*K*_C(21% O2)_] was determined, and the Michaelis constant for oxygenation (*K*_O_) was calculated from the relationship

$$K_{\text{C(21\% O2)}}=K_{\text{C}}\left(1+\begin{array}{c} O_{\text{2}}\\ \bar{K_{O}} \end{array}\right)$$ (3)

Rubisco specificity for CO_2_ over O_2_ (*S*_C/O_) was determined following the method described by Kane *et al.* (1994). Septa-sealed vials containing Rubisco, buffer [30 mM triethanolamine-acetate (pH 8.3), 20 mM Mg-acetate], and 0.2 mg ml^−1^ carbonic anhydrase were incubated in humidified gas (0.1% CO_2_ in O_2_, with a total flow rate of 2000 ml min^−1^; G400 gas mixing system, Qubit Systems, Kingston Canada) at each measurement temperature, with oscillatory shaking. Reactions were initiated by injecting 2 nmol of [^3^H]RuBP (3 kBq nmol^−1^) into the vial and terminated after 60 min by the addition of alkaline phosphatase. To prepare the sample for separation, the reaction products were applied to a 0.5 ml column of BioRad AG1-X8 anion exchange resin (200–400 mesh, formate form), washed with 10 column volumes of ddH_2_O, and radioactively labeled glycerate and glycolate eluted with 10% H_2_SO_4_. The [^3^H]glycerate and [^3^H]glycolate were separated via HPLC (system described in Shay and Kubien, 2013) on an Aminex HPX-87H column (BioRad, Canada) maintained at 60 °C. The mobile phase was 7.5 mM H_2_SO_4_, and the flow rate was 0.4 ml min^−1^. Glycerate and glycolate fractions were collected in drop-synchronization mode (Fraction Collector III, Waters), and the amount of ^3^H in each fraction was determined via scintillation counting. The *S*_C/O_ was calculated from the ratio of [^3^H[glycerate to [^3^H]glycolate and the mole fractions of CO_2_ and O_2_ in the humidified gas, giving *S*_C/O_ expressed as a ratio of partial pressures (Kane *et al.*, 1994). Finally, the average value of each parameter was used to calculate the catalytic rate of oxygenation (*k*_catO2_) from the relationship

$$S_{\text{C/O}}=\frac{k_{\text{catCO2}}}{K_{\text{C}}}\cdot \frac{K_{\text{O}}}{k_{\text{catO2}}}$$ (4)

### MIMS measurement of Rubisco kinetic parameters

Rubisco assays were conducted in a 600 µl temperature-controlled cuvette linked to an isotope ratio mass spectrometer (Thermo-Fischer Delta V) and calibrated as previously described (Cousins *et al.*, 2010; Boyd *et al.*, 2015). Samples were measured similarly to Boyd *et al.* (2015); four oxygen concentrations ranging from 40 μM to 1600 μM, and five CO_2_ concentrations ranging from 0 μM to 200 μM at each oxygen level were made. Measurements were made in 5 °C intervals from 10 °C to 40 °C, and the same three replicates were measured at each temperature. The assay buffer contained 200 mM HEPES (pH 7.7 at each measurement temperature), 20 mM MgCl_2_, 0.1 mM α-hydroxypyridinemethanesulfonic acid (α-HPMS), 8 mg ml^−1^ carbonic anhydrase (Sigma), and 0.6 mM RuBP. A 10 µl aliquot of extract was added per measurement. Rubisco was activated by leaving the extract at room temperature for 10 min prior to returning to ice before measurement.

The measured *v*_c_, *v*_o_, and the corresponding CO_2_ and O_2_ concentrations were fit simultaneously to the following equations

$$v_{\text{c}}=\frac{V_{\text{cmax}}\;CO_{2}}{CO_{2}+K_{\text{C}}(1+O_{2}/K_{\text{O}})}$$ (5)

$$v_{\text{o}}=\frac{V_{\text{omax}}\;O_{2}}{O_{2}+K_{\text{O}}(1+CO_{2}/K_{\text{C}})}$$ (6)

solving for the parameters *V*_cmax_, *V*_omax_, *K*_C_, and *K*_O_. All model fits were performed in the software package Origin 8 (OriginLab) using the non-linear curve-fit function NLfit. *S*_C/O_ was calculated using Equation 4. The *k*_catCO2_ was calculated according to Equation 1 and the *k*_catO2_ was calculated using the analogous equation

$$k_{\text{catO2}}=\frac{V_{\text{omax}}}{\text{active sites}}$$ (7)

It should be noted that plant growth temperature, photoperiod, extraction protocol, and assay conditions were similar but not identical between the MIMS and radiolabel experiments, and, as discussed below, should be taken into account when comparing these two data sets.

### Modeling temperature responses

The temperature responses of the kinetic parameters were calculated for the equation

$$\text{Parameter}=\;k_{25}\;e^{\left(-E_{a}/R\;T_{K})(298.15-T_{K})/(298.15\right)}$$ (8)

where *k*_25_ is the value of the parameter at 25 °C, *E*_a_ is the Arrhenius activation energy (kJ mol^−1^), *R* is the molar gas constant (0.008314 kJ mol^−1^ K^−1^), *T*_K_ is the temperature in Kelvin, and the term (298.15–*T*_K_)/298.15 is the scaling factor so that *k*_25_ may be used as the pre-exponential term. The *E*_a_ and *k*_25_ values for each Rubisco parameter were calculated by a linear regression of the natural log of the data plotted against (*T*_K_–298.15)/(*T*_K_), such that the *y*-intercept was equal to the natural log of *k*_25_ and the slope was equal to *E*_a_/(298.15 *R*). For comparison, the non-transformed temperature responses are presented in Supplementary Fig. S1 and Supplementary Table S2. Three replicates of *E*_a_ and *k*_25_ were determined for each parameter, with the exception of radiolabel *S*_C/O_ where the number of replicates was four. For all MIMS and radiolabel comparisons, other than *k*_catCO2_, only the curve fitting methods are compared. For simplicity, we exclude the radiolabel single point when comparing ratios of kinetic parameters with MIMS. Differences in the *k*_25_ and *E*_a_ values were determined by ANOVA, followed by pair-wise comparison (Tukey HSD) with a significance cut-off of *P*<0.05 in Statistix 9 (Analytical Software, Tallahassee, FL, USA).

Arrhenius plots for all kinetic parameters were examined for thermal breaks using the package ‘segmented’ in R, which first tests for differences between slopes using the Davies test (Davies, 1987), and then estimates the breakpoints in linear models using maximum likelihood (Muggeo, 2003, 2008; R Core Team, 2013). When breakpoints in the Arrhenius temperature response plots were statistically valid, the *E*_a_ values above and below the break points were compared with other *E*_a_ values as described above; the *k*_25_ value was held constant when fitting for two *E*_a_ values above and below the breakpoint.

### Equations for reaction mechanisms

Figure 1 depicts the currently hypothesized reaction mechanism of Rubisco as originally described by Farquhar (1979). The kinetic parameters *k*_catCO2_, *k*_catO2_, *K*_C_, *K*_O_, and *S*_C/O_ can be described by the individual first-order rate constants (*k*) seen in Fig. 1 as follows:

$$k_{\text{catCO2}}=\frac{k_{8}k_{9}}{k_{8}+k_{9}}$$ (9)

$$k_{\text{catO2}}=\frac{k_{5}k_{9}}{k_{5}+k_{9}}$$ (10)

$$K_{\text{C}}=\frac{k_{7}+k_{8}}{k_{6}}\frac{k_{9}+k_{10}}{k_{8}+k_{9}}\approx k_{\text{catCO2}}\frac{k_{9}+k_{10}}{k_{9}k_{6}}$$ (11)

$$K_{\text{O}}=\frac{k_{4}+k_{5}}{k_{3}}\frac{k_{9}+k_{10}}{k_{5}+k_{9}}\approx k_{\text{catO2}}\frac{k_{9}+k_{10}}{k_{9}k_{3}}$$ (12)

$$S_{\text{C/O}}=\frac{k_{6}}{k_{3}}\frac{k_{4}+k_{5}}{k_{7}+k_{8}}\frac{k_{8}}{k_{5}}\approx \frac{k_{6}}{k_{3}}$$ (13)

where the subscript indicates the transition state as numbered in Fig. 1 by the black circles. The approximations in Equations 11–13 are made by assuming that the rates of decarboxylation (*k*_7_) and deoxygenation (*k*_4_) are negligible.

These first-order rate constants can be related to temperature using transition state theory and the Eyring equation

$$k=\frac{k_{\text{B}}T}{h}e^{-\Delta G^{‡}/RT}$$ (14)

where *k*_B_ is the Boltzmann constant (1.3807 × 10^–23^ J K^−1^), *h* is the Planck constant (6.6261 × 10^–34^ J s), and Δ*G*^‡^ (J mol^−1^) is the standard free energy difference between the transition state and the substrate (or intermediate). Note that the proportionality constant *κ*, describing the proportion of vibrations that lead to product formation, has been assumed equal to one and left out of the equation. The Δ*G*^‡^ has components of entropy (Δ*S*^‡^) and enthalpy (Δ*H*^‡^) as defined by

$$\Delta G^{‡}=\Delta H^{‡}-T\Delta S^{‡}$$ (15)

where the double dagger symbol (^‡^) denotes the transition state.

### Modeling rate constants (*k*) and Δ*G*^‡^

The proposed Rubisco reaction mechanism (Fig. 1) suggests that *k*_catCO2_, *k*_catO2_, *K*_C_, *K*_O_, and *S*_C/O_ are described by complex terms made up of two or more elementrary reaction rates (Farquhar, 1979; Equations 9–13). The rate constant (*k*) is related to the energy barrier for the transition state of the reaction, often referred to as the activation energy (Δ*G*^‡^). The relationship between *k*, Δ*G*^‡^, and temperature is described by the Eyring equation (Equation 14), where Δ*G*^‡^ has enthalpic (Δ*H*^‡^) and entropic (Δ*S*^‡^) components (Equation 15). From Equation 15, a plot of Δ*G*^‡^ with temperature has a slope of Δ*S*^‡^ and a *y*-intercept of Δ*H*^‡^. For the discussion of Rubisco kinetics, all numbering of *k*, Δ*G*^‡^, Δ*H*^‡^, and Δ*S*^‡^ refers to reaction steps initially described by Farquhar (1979) and reproduced in Fig. 1. The Eyring equation has been previously used to calculate Δ*G*^‡^ values for *k*_catCO2_, *k*_catO2_, and *S*_C/O_ (Chen and Spreitzer, 1992; Tcherkez *et al.*, 2006; McNevin *et al.*, 2007; Tcherkez, 2013). Because *k*_catCO2_ and *k*_catO2_ are first-order rate constants, they have been represented as

$$-\ln\left(k_{\text{catCO2}}\frac{h}{k_{\text{B}}T}\right)RT=\Delta G_{\text{kcatCO2}}^{‡}=\Delta H_{\text{kcatCO2}}^{‡}-T\Delta S_{\text{kcatCO2}}^{‡}$$ (16)

and

$$-\ln\left(k_{\text{catO2}}\frac{h}{k_{\text{B}}T}\right)RT=\Delta G_{\text{kcatO2}}^{‡}=\Delta H_{\text{kcatO}2}^{‡}-T\Delta S_{\text{kcatO2}}^{‡}$$ (17)

and because *S*_C/O_ is the ratio of two first-order rate constants (Equation 13), it has been represented as

$$\ln\left(S_{\text{C/O}}\right)RT=\Delta G_{3}^{‡}-\Delta G_{6}^{‡}=(\Delta H_{3}^{‡}-\Delta H_{6}^{‡})-T(\Delta S_{3}^{‡}-\Delta S_{6}^{‡})$$ (18)

The Δ*G*^‡^ terms in Equations 16–18 can be calculated directly from measured values, and the Δ*H*^‡^ and Δ*S*^‡^ terms would describe a linear fit of Δ*G*^‡^ to the temperature response, assuming Δ*H*^‡^ and Δ*S*^‡^ are constant within the temperature range. However, the use of Equations 16–18 does not provide information regarding an elementary rate constant or a corresponding energy barrier. Modeling to estimate individual rate constants from the measured data is described below.

#### Modeling of radiolabel data

Each of the rate constants (*k*) in Fig. 1 has a corresponding energy of activation (Δ*G*^‡^ from Equation 14), which has a corresponding enthalpic and entropic component (Δ*H*^‡^ and Δ*S*^‡^ from Equation 15). We assumed that the values of Δ*H*^‡^ and Δ*S*^‡^ are constant within the temperature range. Therefore, we fit Michaelis–Menten parameters calculated from elementary rate constants using Equations 9–13 to the measured Michaelis–Menten parameters by varying the corresponding Δ*H*^‡^ and Δ*S*^‡^ values. All modeling used the solver function in Excel (2016, Microsoft, Redmon, WA, USA) to minimize the sum of the differences squared between modeled and measured parameters.

The rate constants *k*_8_ (cleavage of carboxylated intermediate) and *k*_9_ (enolization of RuBP) were calculated from measured *k*_catCO2_ values following the calculations of Tcherkez *et al.* (2013) such that *k*_8_/*k*_9_ is 0.83 at 25 °C. The rate constant *k*_10_ (de-enolization) was modeled assuming *k*_9_/*k*_10_ is 0.43 at 25 °C following the calculations of Tcherkez *et al.* (2013); we further assumed that this ratio remained constant with temperature. This allowed for calculation of the rate of *k*_6_ (CO_2_ addition) as the only remaining unknown when fitting measured values of *K*_C_ with Equation 11 assuming *k*_7_ (de-carboxylation) was negligible. After calculating *k*_6_, then *k*_3_ (O_2_ addition) was modeled from measured *S*_C/O_ values and Equation 13, assuming rate constants *k*_7_ (decarboxylation) and *k*_4_ (deoxygenation) are negligible. Finally, the rate constant *k*_5_ (cleavage of the oxygenated intermediate) was calculated from measured *K*_O_ values and Equation 14, again assuming *k*_4_ (deoxygenation) was negligible. This process allowed for estimation of the temperature response for *k* and Δ*G*^‡^ values for each step of the reaction mechanism listed in Equations 9–13, with the exception of the decarboxylation and deoxygenation steps that were assumed to be negligible (Tcherkez *et al.*, 2013; Tcherkez, 2013, 2016).

#### Modeling of MIMS data

For the MIMS data, where measurements of *k*_catO2_ were available and non-linearity of Arrhenious plots was observed, the rate constants and corresponding Δ*G*^‡^, Δ*H*^‡^, and Δ*S*^‡^ values were determined differently from what was described above for the radiolabel data. The Δ*H*^‡^ and Δ*S*^‡^ values corresponding to the rate constants for *k*_8_ (cleavage of carboxylated intermediate), *k*_5_ (cleavage of oxygenated intermediate), and *k*_9_ (RuBP enolization) were determined by fitting to measured *k*_catCO2_ and *k*_catO2_ values, assuming *k*_8_/*k*_9_ was 0.83 at 25 °C, and using Equations 9 and 10. Because *k*_catCO2_ showed a breakpoint, it is possible that *k*_8_ and *k*_9_ have different temperature responses, with a crossover at ~25 °C. However, *k*_catO2_ also showed a breakpoint at 25 °C and the carboxylated intermediate cleavage rate (*k*_8_) is much greater than the oxygenated cleavage rate (*k*_5_) because measured *k*_catCO2_ values are greater than measured *k*_catO2_. Therefore, a crossover of *k*_5_, *k*_8_, and *k*_9_ at a single temperature is not possible and a breakpoint in *k*_catCO2_ and *k*_catO2_ co-occuring at a single temperature cannot be modeled as a changing rate-limiting step. Therefore, we modeled the breakpoint in *k*_catO2_ by allowing *k*_5_ to have separate Δ*H*^‡^ and Δ*S*^‡^ values above and below the breakpoint, and assuming *k*_9_ had the same values of Δ*H*^‡^ and Δ*S*^‡^ above and below the breakpoint. Because *k*_9_ (rate constant of RuBP enolization) temperature response was assumed constant for models of *k*_catO2_, it was also assumed constant when modeling *k*_catCO2_. Therefore, *k*_8_ was allowed to have separate values of Δ*H*^‡^ and Δ*S*^‡^ above and below the breakpoint. The *k*_10_ (rate constant of de-eneolization) was subsequently calculated assuming the ratio *k*_9_/*k*_10_ was 0.43 and constant with temperature. The value *k*_6_ (rate constant of CO_2_ addition) was then calculated from measured *K*_C_ and the approximation of Equation 11 assuming decarboxylation is negligible. This was also done for *k*_3_ (rate constant for O_2_ addition) using *K*_O_ and the approximation of Equation 12 assuming de-oxygenation (*k*_4_) was negligable. It was required that *k*_6_ and *k*_3_ have seperate Δ*H*^‡^ and Δ*S*^‡^ values above and below the breakpoint to model linear Arrhenious plots of *K*_C_ and *K*_O_. This process provided estimates of the temperature response for *k* and Δ*G*^‡^ values for each step of the reaction mechanisms making up the measured Michaelis–Menten parameters (Equations 9–13), with the exception of the decarboxylation and deoxygenation steps, which were assumed to be negligable.

## Results

### Breakpoints

**Fig. 2.**
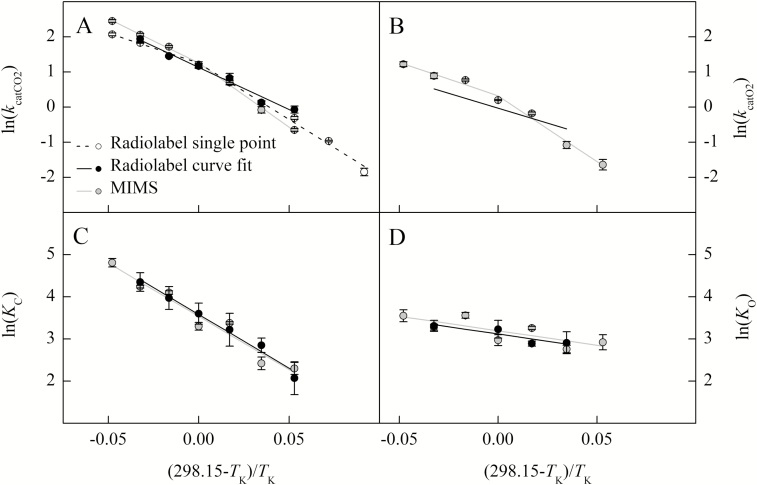
The natural log of Rubisco parameters from *Arabidopsis thaliana* measured using radiolabel (single point, open circle; curve fit, black circle) and MIMS (gray circle) methods are plotted against the inverse of the temperature in Kelvin offset to a *y*-intercept of 25 °C. The temperature response of catalytic turnover for CO_2_ (*k*_catCO2_, s^−1^, A) and O_2_ (*k*_catO2_, s^−1^, B), and the Michaelis–Menten constant for CO_2_ (*K*_C_, Pa, C) and O_2_ (*K*_O_, kPa, D) are shown. The lines represent the model fit to the measured data. The radiolabel *k*_catO2_ model in (B) was determined from the relationship with *S*_C/O_ described by Equation 4.

**Fig. 3.**
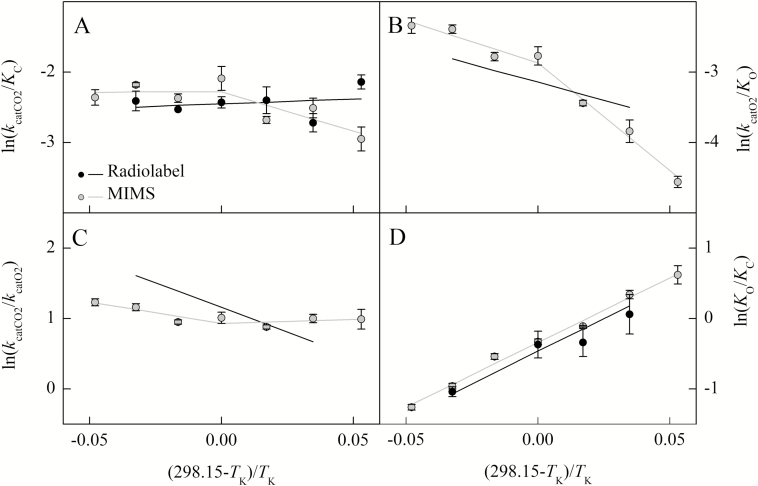
The natural log of the Rubisco parameter ratios from *Arabidopsis thaliana* measured using radiolabel (black circle) and MIMS (gray circle) are plotted against the inverse of the temperature in Kelvin offset to a *y*-intercept of 25 °C. The temperature response of the catalytic efficiency of the carboxylation (*k*_catCO2_/*K*_C_, A) and oxygenation (*k*_catO2_/*K*_O_, B) reactions, catalytic turnover ratio for CO_2_ over O_2_ (*k*_catCO2_/*k*_catO2_, C), and the Michaelis–Menten constant ratio for O_2_ over CO_2_ (*K*_O_/*K*_C_, D) are shown. Lines represent the combination of models represented in Fig. 2 and are not the result of linear regressions to the ratios.

**Fig. 4.**
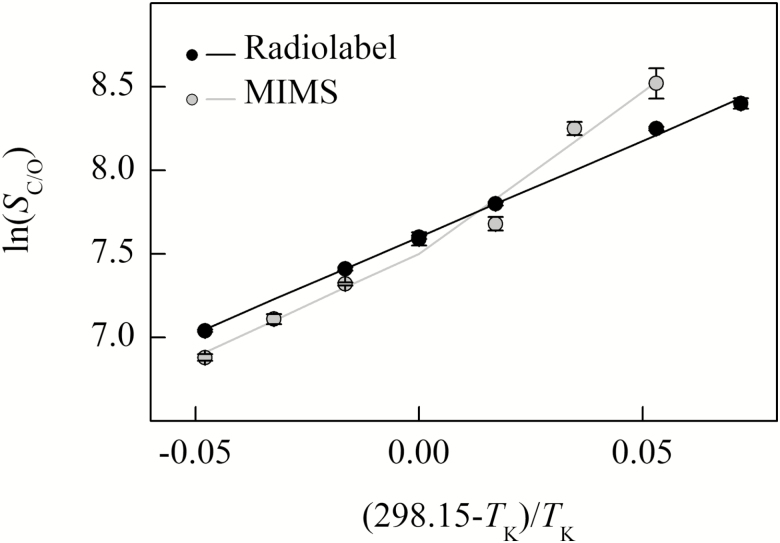
The natural log of Rubisco specificity for CO_2_ over O_2_ (*S*_C/O_) from *Arabidopsis thaliana* measured using radiolabel (black circle) and MIMS (gray circle) methods are plotted against the inverse of the temperature in Kelvin offset to a *y*-intercept of 25 °C. The black line represents the model fit to the measured radiolabel values. The gray line was determined from the relationship of *S*_C/O_ to the parameters presented in Fig. 2, described by Equation 4.

The Davies test indicated significant breakpoints for the *k*_catCO2_, *k*_catO2_, and *S*_C/O_ temperature response for the MIMS data as well as for the radiolabel single point measurement of *k*_catCO2_ (Table 1; Figs 2, 4). Both the Davies test and the maximum likelihood segmented analysis indicated that the breakpoints in these parameters were near 25 °C (Table 1). All other parameters showed no breakpoints in their temperature responses for either the MIMS or radiolabel data sets (Table 1; Figs 2–4).

**Table 1.** Testing for thermal breaks for all kinetic parameters

Method	Parameter	Davies test		Maximum likelihood		
		Estimated breakpoint (°C)	*P*-value	Estimated breakpoint (°C)	*CI* (lower)	CI (upper)
Radiolabel	*k*_catCO2_ single point	26.8	*	25.1	5.3	36.9
	*k*_catCO2_ curve fit	–	ns			
	*k*_catO2_	–	–			
	*K*_C_	–	ns			
	*K*_O_	–	ns			
	*S*_C/O_	–	ns			
MIMS	*k*_catCO2_	25.3	*	25.3	23.1	31.5
	*k*_catO2_	25.3	*	25.5	24.3	32.6
	*K*_C_	–	ns			
	*K*_O_	–	ns			
	*S*_C/O_	25.4	*	25.2	15.0	27.6

Arrhenius plots were examined using the package ‘segmented’ in R (R Core Team, 2013), which determines the significance of breakpoints in linear models and estimates breakpoint locations as described by Davies (1987). Additionally, breakpoint locations and confidence intervals (CIs, lower and upper) were independently estimated using a maximum likelihood test (Muggeo, 2003, 2008). * indicates a *P*-value <0.05 for the Davies test and ns is not significant.

### Arrhenius activation energies and modeled value at 25 °C

The modeled 25 °C values (*k*_25_) and Arrhenius activation energy (*E*_a_) above 25 °C agree with many of the literature values for other C_3_-type Rubisco, including *in vitro* and *in vivo* measurements of *A. thaliana* (Flexas *et al.*, 2007; Whitney *et al.*, 2011; Walker *et al.*, 2013; Weise *et al.*, 2015; Galmés *et al.*, 2016). Although, previous reports of Rubisco specificities for CO_2_ over O_2_ (*S*_C/O_) at 25 °C vary widely for C_3_ species, including for *A. thaliana* which range from below 2125 to above 2655 (Pa Pa^−1^; Flexas *et al.*, 2007; Whitney *et al.*, 2011; Walker *et al.*, 2013; Weise *et al.*, 2015). For the MIMS-derived parameters with breakpoints (*k*_catCO2_, *k*_catO2_, and *S*_C/O_), and the radiolabel single point estimate of *k*_catCO2_, the lower temperature *E*_a_ values were larger than *E*_a_ values estimated at higher temperatures (Tables 2, 3). Above 25 °C, the *E*_a_ values were similar for all parameters between the radiolabel and MIMS curve fitting methods. The radiolabel *E*_a_ for *k*_catCO2_ determined by curve fitting across all temperatures was intermediate to the two *E*_a_ values estimated above and below the breakpoint from the single point radiolabel data. The *k*_25_ values for *k*_catCO2_ estimated from radiolabel and MIMS methods were not different from each other, but were larger than the *k*_25_ for *k*_catO2_ determined by MIMS (Table 2). The *E*_a_ and *k*_25_ values for *K*_C_ and *K*_O_ were not significantly different between methods (Table 3). However, the MIMS *S*_C/O_ measured from 10 °C to 25 °C had a lower (more negative) *E*_a_ value than the MIMS *S*_C/O_*E*_a_ value measured from 25 °C to 40 °C and the radiolabel *S*_C/O_*E*_a_ value (Table 3).

**Table 2.** Comparison of *k*_25_ and *E*_a_ values for *k*_cat_ measurements from the different methods

Method	Temperature (°C)	Parameter	*k*_25_	*E*_a_
Radiolabel				
Single point	0–25	*k*_catCO2_ (s^−1^)	3.50 ± 0.20 A	79.53 ± 2.03 a
	25–40		–	42.11 ± 3.45 c
Curve fit	10–35		3.10 ± 0.07 A	59.64 ± 3.93 b
MIMS	10–25		3.53 ± 0.25 A	90.36 ± 1.03 a
	25–40		–	62.20 ± 2.68 b
	10–25	*k*_catO2_ (s^−1^)	1.38 ± 0.05 B	92.95 ± 7.31 a
	25–40		–	47.11 ± 2.33 b,c

The *k*_25_ and *E*_a_ values are the mean of 3–4 replicates, calculated from linear regressions of Arrhenius plots. The temperature ranges for each regression were determined by segment analysis. Letters indicate significant differences between groups (Tukey HSD, *P*<0.05).

**Table 3.** Comparison of *K*_C_, *K*_O_, *S*_C/O_ parameters *k*_25_ and *E*_a_ resulting from the different methods

Method	Temperature range (°C)	Parameter	*k*_25_ (Pa)	*E*_a_ (kJ mol^−1^)
Radiolabel	10–35	*K*_C_	36 ± 2	63.09 ± 6.23
MIMS	10–40		34 ± 1	62.62 ± 3.44
Radiolabel	15–35	*K*_O_	23 100 ± 3430	16.89 ± 2.59
MIMS	10–40		24 400 ± 701	17.01 ± 2.48
Radiolabel	05–40	*S*_C/O_	2003 ± 22	–28.66 ± 0.51 b
MIMS	10–25		1814 ± 117	–48.19 ± 4.17 a
	25–40		–	–30.51 ± 6.41 b

No differences were observed in *k*_25_ between methods. No differences were observed in *E*_a_ values for *K*_C_ and *K*_O_ values between methods (ANOVA). Theletters next to the *E*_a_ values indicate significant differences for the *S*_C/O_ values (Tukey HSD, *P*<0.05).

The *E*_a_ value for the carboxylation efficiency (*k*_catCO2_/*K*_C_) below 25 °C was significantly different from zero for the MIMS method, where the carboxylation efficiency increased with temperature; however, above 25 °C, the *E*_a_ value was not significantly different from zero (Table 4). The MIMS *E*_a_ for oxygenation efficiency (*k*_catO2_/*K*_O_) was significantly different from zero above and below 25 °C (Table 4). The *E*_a_ for the ratio of catalytic rates (*k*_catCO2_/*k*_catO2_) measured by MIMS was only significantly different from zero above 25 °C (Table 4). The *E*_a_ for *K*_O_/*K*_C_ was significantly different from zero for both radiolabel and MIMS methods (Table 4).

**Table 4.** The *E*_a_ and *k*_25_ parameters for *k*_catCO2_/*K*_C_, *k*_catO2_/*K*_O_, *k*_catCO2_/*k*_catO2_, and *K*_O_/*K*_C_ ratios

Method	Temperature range (°C)	Parameter	*k*_25_	*E*_a_
Radiolabel	10–35	*k*_catCO2_/*K*_C_	0.09 ± 0.00	–3.45 ± 3.94
MIMS	10–25	(s^−1^ Pa^−1^)	0.10 ± 0.01	27.75 ± 3.38*
	25–40		–	–0.41 ± 6.10
MIMS	10–25	*k*_catO2_/*K*_O_	0.06 ± 0.00	75.93 ± 7.41*
	25–40	(s^−1^ kPa^−1^)	–	30.09 ± 0.70*
MIMS	10–25	*k*_catCO2_/*k*_catO2_	2.55 ± 0.16	–2.58 ± 6.73
	25–40		–	15.10 ± 4.92*
Radiolabel	15–35	*K*_O_/*K*_C_	0.65 ± 0.11	–46.20 ± 8.80*
MIMS	10–40	(kPa Pa^−1^)	0.71 ± 0.01	–45.60 ± 2.57*

The *E*_a_ parameters were tested to determine if they were significantly different from zero (*t*-test), where the * next to the *E*_a_ values indicates a *P*-value <0.05.

### Modeling *k* and Δ*G*^‡^

**Fig. 5.**
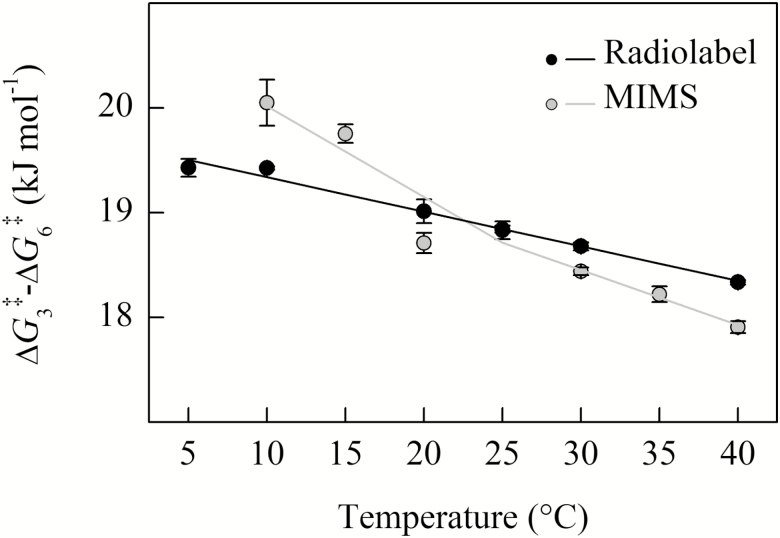
The temperature response of Δ*G*_3_^‡^–Δ*G*_6_^‡^ calculated from the data presented in Fig. 4. Both measurement methods show a decrease with temperature. Solid black circles are the mean of four replicates measured using radiolabel, filled gray circles are the means from three replicates using MIMS; the SE is shown. The solid lines indicate the linear regression fit to calculated values.

Above 25 °C, the Δ*G*_3_^‡^–Δ*G*_6_^‡^ for *S*_C/O_ from radiolabel and MIMS (Fig. 5) are similar to previous calculations for C_3_ species reported by Tcherkez *et al.* (2006). However, the MIMS entropy difference between O_2_ and CO_2_ addition (Δ*S*_3_^‡^–Δ*S*_6_^‡^, slope of the line in Fig. 5; see Equation 18; see Supplemenary Table S3) from data colleted below 25 °C appear more similar to the Δ*S*_3_^‡^–Δ*S*_6_^‡^ of red algae rather than of higher plants, when compared with data presented in Tcherkez *et al.* (2006).

**Fig. 6.**
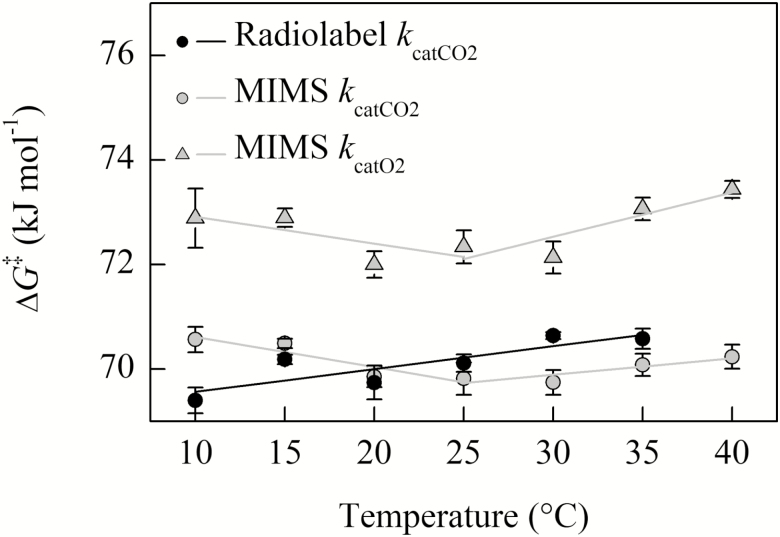
The temperature response of Δ*G*_kcatCO2_^‡^ for MIMS and radiolabel methods, and Δ*G*_kcatO2_^‡^ for MIMS calculated from the data presented in Fig. 2. Two regressions were fit to the MIMS data on either side of the 25 °C breakpoint; a single regression is fit to the radiolabel data. Solid black circles are the mean of three replicates measured using radiolabel, filled gray circles are the means from three replicates using MIMS; the SE is shown.

The free energy of activation associated with *k*_catCO2_ (Δ*G*_kcatCO2_^‡^) plotted against temperature increased linearly for the radiolabel curve fit method, while the Δ*G*_kcatCO2_^‡^ calculated from MIMS measurements decreased from 10 °C to 25 °C and then increased from 25 °C to 40 °C (Fig. 6). A similar temperature response was also observed for MIMS Δ*G*_kcatO2_^‡^, although the absolute values of Δ*G*_kcatO2_^‡^ are larger than Δ*G*_kcatCO2_^‡^ as evident by a lower *k*_catO2_ compared with *k*_catCO2_ at all temperatures (i.e. larger energy barriers result in slower reactions). The slope of Δ*G*_kcatCO2_^‡^ values presented in Fig. 6 (equivalent to the entropy term Δ*S*_kcatCO2_^‡^; see Supplementary Table S4) calculated for radiolabel and MIMS above 25 °C are slightly larger than those reported for *Nicotiana tabacum* (McNevin *et al.*, 2007). The MIMS Δ*S*_kcatCO2_^‡^ and Δ*S*_kcatO2_^‡^ showed a sign change above and below the breakpoint (negative slope to positive slope, Fig. 6; Supplementary Table S4).

**Fig. 7.**
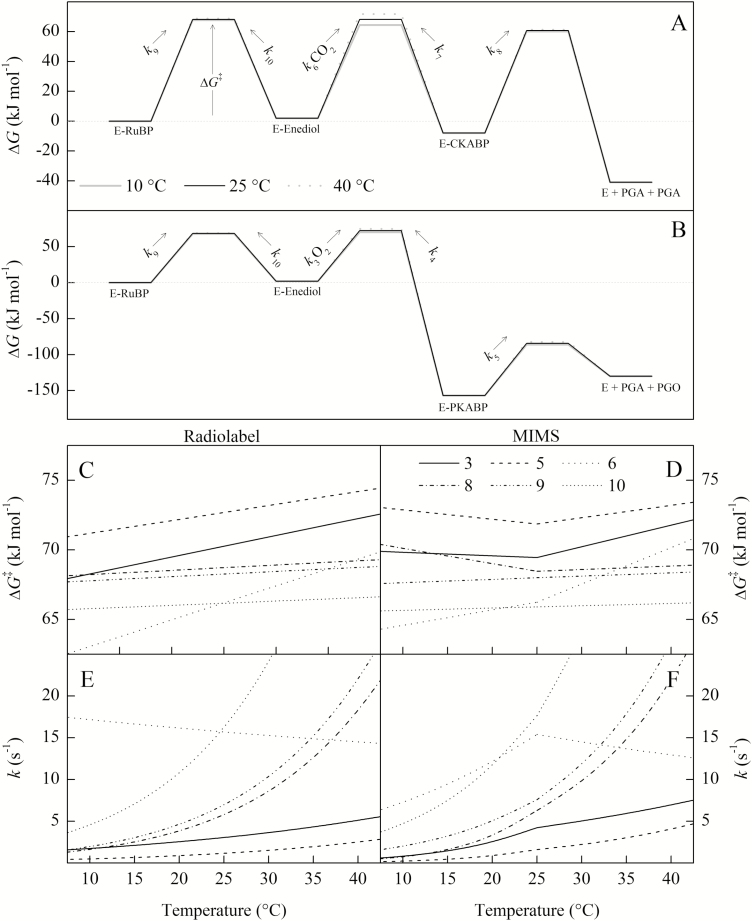
A kinetic energy barrier diagram showing the modeled temperature responses of the energy barrier to the transition state (Δ*G*^‡^) and the corresponding first-order rate constant *k*. The Δ*G*^‡^ and *k* are indicated by the numbered step of the reaction following Fig. 1. The assumptions made for this model are stated in the Materials and methods. For steps 3 and 6 (O_2_ and CO_2_ addition, respectively), the rate constants were multiplied by ambient concentrations O_2_ (21 kPa) and CO_2_ (41 Pa) as a pseudo-first-order approximation for comparison with the other rate constants and to calculate their respective Δ*G*^‡^. For the bottom figure, the left-hand column is modeled on the radiolabel data and the right-hand column on the MIMS data so that comparisons between continuous and breakpoint temperature responses can be made. The values for intermediates were taken from Tcherkez (2013) for (A) and Tcherkez (2016) for (B) and assumed to remain constant with temperature.

Temperature responses of the rate constants (*k*) and corresponding energy barriers of the transition states (Δ*G*^‡^) are shown in Fig. 7, while the modeled Δ*H*^‡^ and Δ*S*^‡^ values are presented in Suppementary Table S5. Calculations of elementary rate constants and corresponding Δ*G*^‡^ are similar to previous calculations at 25 °C from Tcherkez (2013, 2016). In order to model breakpoints in the MIMS *k*_catCO2_, *k*_catO2_, and *S*_C/O_ parameters, breakpoints are neeeded in the rate constants for the cleavage (*k*_8_ and *k*_5_) and for gas addition (*k*_6_ and *k*_3_). This is required because it was not possible to model a simultaneous change in the rate-limiting step for both the *k*_catCO2_ and *k*_catO2_ parameter (Supplementary Fig. S2). This further required that breakpoints were needed in the rate constants for CO_2_ and O_2_ addition (*k*_6_ and *k*_3_, respectively) to maintain the observed linearity for the *K*_C_ and *K*_O_ Arrhenius plots (Fig. 2).

## Discussion

### Radiolabel single point *k*_catCO2_ breakpoint

The radiolabel single point method reported here utilized a single bicarbonate concentration with temperature (11 mM) and resulted in a thermal breakpoint similar to Björkman and Pearcy (1970). Because Björkman and Pearcy (1970) suggested that there could be inhibition at low temperature and subsaturating concentrations at high temperature, we plotted the predicted CO_2_ concentration achieved by 11 mM NaHCO_3_ at each temperature against the measured and modeled CO_2_ response of the enzyme determined by both radiolabel and MIMS curve fitting methods (Supplementary Fig. S3). The CO_2_ concentration provided by the 11 mM NaHCO_3_ appears saturating at 10 °C and 15 °C, but becomes increasingly less saturating at higher temperatures, as indicated where the shaded area intersects the modeled CO_2_ response (Supplementary Fig. S3). This suggests that the lower *E*_a_ value of the single point method at high temperatures could be caused by subsaturating CO_2_ concentrations.

### MIMS *k*_catCO2_, *k*_catO2_, and *S*_C/O_ breakpoints

The non-linearity of Arrhenius plots of *k*_catCO2_, *k*_catO2_, and *S*_C/O_ for the MIMS data were interpreted as 25 °C breakpoints. Badger and Collatz (1977) also observed breakpoints in *k*_catCO2_, *k*_catO2_, and *S*_C/O_; however, they observed an additional thermal breakpoint in *K*_C_, which was not observed with the MIMS data presented here. As *S*_C/O_ is a ratio of *k*_catCO2_, *K*_C_, *K*_O_, and *k*_catO2_ (Equation 4), the differences in *S*_C/O_ breakpoints between Badger and Collatz (1977) and our MIMS data could suggest different mechanisms driving the thermal response of *S*_C/O_. Furthermore, no breakpoint in *S*_C/O_ has been observed in any study using the [^3^H]RuBP method.

The breakpoints observed in MIMS *k*_catCO2_ and *k*_catO2_ are unlikely to be caused by insufficient or inhibitory CO_2_ concentrations, as subsaturation or inhibition should be evident in the CO_2_ response curves (Supplementary Fig. S3). A breakpoint in both *k*_catCO2_ and *k*_catO2_ could be caused by deactivation of the enzyme, as was suggested by Kubien *et al.* (2003). However, deactivation is unlikely to change the *k*_catCO2_/*k*_catO2_ temperature response as was observed in Fig. 3C, because both catalytic rates are expected to be affected in the same way by deactivation. Alternatively, the observed breakpoints in MIMS could be related to methodology as the radiolabel Arrhenius plots presented here for *k*_catCO2_ and *S*_C/O_ were sufficiently linear.

### Limitations of methodological comparisons

The Rubisco kinetic parameters for *A. thaliana* measured with the radiolabel and MIMS curve fitting methods were similar at and above 25 °C, suggesting similar kinetic parameters under these conditions, despite slight differences in plant growth environments, as well as sample extraction and assay conditions. However, at lower temperatures, the observed breakpoints in MIMS and the corresponding linearity of the Radiolabel temperature responses could imply that plant-specific growth differences were important. For example, spinach Rubisco appears to acclimate to growth temperature, with warm-grown Rubisco showing a thermal breakpoint in the carboxylation rate at 15 °C, below which rates are lower than those of a cold-grown enzyme (Yamori *et al.*, 2006). This is similar to the breakpoint evident in the MIMS data set presented here; however, the daytime temperature differential between plants grown for the MIMS (23 °C) and radiolabel (20 °C) plants was much smaller than the 15 °C differential used by Yamori *et al.* (2006). Further, the MIMS technique had a lower *S*_C/O_ than radiolabel parameters at temperatures above 25 °C, and a higher value at temperatures below 25 °C, opposite to what Yamori *et al.* (2006) observed, suggesting that the kinetic differences between the MIIMS and radiolabel measurements were not due to temperature acclimation of Rubisco.

The possibility remains that the differences, particularly at cold temperatures, are due to methodology artifacts arising from differences in buffer composition. However, preparations of Rubisco for MIMS or radiolabel assays both include components known to affect Rubisco stability (i.e. DTT, MgCl_2_, and NaHCO_3_), albeit at different concentrations. It is also possible that either the MIMS or the radiolabel assays causes erroneous kinetic estimates at low temperatures; however, this uncertainty is difficult to explain given that breakpoints have been observed by different laboratories using varying methods and species (Badger and Collatz, 1977; Sage, 2002, Kubien *et al.*, 2003; Sharwood *et al.*, 2016). Therefore, additional analysis of diverse species with the MIMS system is needed to better understand if this is a technique- or species-specific phenomenon.

Nevertheless, breakpoints have persisted in the Rubisco literature for >40 years without sufficient explanation and warrant further investigations into their underlying causes. Badger and Collatz (1977) suggested that changes in the rate-limiting step of the reaction mechanism were brought about by conformational changes. If the elementary rate constants defining a specific parameter have different temperature responses then this could cause breakpoints if they cross over, causing a change in the rate-limiting step. The discussion below utilizes the currently accepted reaction mechanism of Rubisco (Fig. 1) and transition state theory to explore breakpoints as a function of changes in energy barriers to elementary reactions.

### Rubisco reaction mechanisms and breakpoints

For the MIMS data, the breakpoints observed in *k*_catCO2_ and *k*_catO2_ could be due to changes in the rate-limiting step, as suggested by Badger and Collatz (1977). For example, *k*_catCO2_ is a function of the rate of cleavage of the carboxylated intermediate (*k*_8_) and the rate of RuBP enolization (*k*_9_). This would mean that *k*_8_ and *k*_9_ have different a temperature response such that they cross over at around the breakpoint observed at 25 °C. However, modeling this change in rate-limiting steps due to different temperature responses cannot simultaneously explain the observed breakpoint in *k*_catCO2_ and *k*_catO2_, because the value of *k*_5_ defining the cleavage of the oxygenated intermediate is lower than *k*_8_. This means that *k*_9_ cannot cross over both *k*_8_ and *k*_5_ at 25 °C (Supplementary Fig. S2).

In order to model the reaction mechanism suggested by MIMS measurements, breakpoints in four elementary rate constants (*k*_3_, *k*_5_, *k*_6_, and *k*_8_) are needed to describe the breakpoints in *k*_catCO2_, *k*_catO2_, and *S*_C/O_ (Fig. 7D, E). While it seems unlikely that such an entropy change could be driven by a conformation change in the enzyme brought about by such minimal changes in temperature, a similar change in entropy for *k*_catCO2_ was observed between wild-type *N. tabacum* and a mutant (L335V) Rubisco (McNevin *et al.*, 2007). This could suggest that the entropy changes proposed here may be possible given enzyme conformational changes with temperature.

The modeling presented here is largely based on isotope exchange studies, which suggest similar energy barriers between enolization (Δ*G*_9_^‡^) and cleavage (Δ*G*_8_^‡^). However, these measurements have been limited to 25 °C (Van Dyk and Schloss, 1986; Tcherkez *et al.*, 2013), and extension of isotope exchange studies to temperature responses would help constrain how the elementary rate constants vary with temperature. Contrary to the above proposal that the cleavage transition state (*k*_8_) undergoes changes above and below 25 °C, is that Rubisco discrimination against ^13^CO_2_ is believed to remain constant with temperature (Christeller and Laing, 1976). If the rate of cleavage (*k*_8_) decreases, then the decarboxylation reaction (*k*_7_) may increase, or the *k*_7_/*k*_8_ ratio could increase, which would change Rubisco discrimination against ^13^CO_2_. Furthermore, the above modeling relies on the assumption that decarboxylation (*k*_7_) was negligible at all temperatures; therefore, changes in fractionation with temperature for an enzyme exhibiting breakpoints should help test the validity of these assumptions.

### Conclusion

The measured temperature responses of Rubisco kinetic parameters were consistent between methods at and above 25 °C; however, there were thermal breakpoints at 25 °C in the MIMS data set for *k*_catCO2_, *k*_catO2_, and *S*_C/O_. Additionally, the radiolabel method using a single bicarbonate concentration showed a breakpoint for *k*_catCO2_ probably caused by non-saturating CO_2_ concentrations at higher temperatures. Previous studies suggest that breakpoints are caused by either a change in the rate-limiting step of the reaction mechanism or deactivation of the enzyme at low temperatures. By modeling elementary steps of the reaction mechanism, we showed that neither cause is sufficient to explain simultaneous breakpoints in *k*_catCO2_, *k*_catO2_, and *S*_C/O_. Instead, breakpoints in the elementary rate constants would be needed. Because the modeling presented here is largely based on isotope exchange studies, moving forward, the temperature response of isotopic substitution experiments would advance our understanding of how elementary rate constants change in relation to one another with temperature.

## Supplementary data

Supplementary data are available at *JXB* online.

Fig. S1. Temperature response of Rubisco parameters from *Arabidopsis thaliana* measured using radiolabel and MIMS methods.

Fig. S2. Two possible crossover models that result in breakpoints for *k*_catCO2_ for MIMS data.

Fig. S3. CO_2_ response curves from 10 °C to 40 °C showing measured values from the radiolabel and MIMS curve fitting methods.

Table S1. p*K*a values used in calculations.

Table S2. Average Rubisco kinetic parameters measured at each temperature

with ±SE.

Table S3. The Δ*H*^‡^ and Δ*S*^‡^ calculated for the Δ*G*^‡^ values presented in Fig. 5 using Equation 18.

Table S4. The Δ*H*^‡^ and Δ*S*^‡^ calculated for the Δ*G*^‡^ values presented in Fig. 6 using Equations 16 and 17.

Table S5. The Δ*H*^‡^ and Δ*S*^‡^ calculated for the Δ*G*^‡^ values presented in Fig. 7 using Equations 9–15.

Supplementary Figures S1-S3 Tables S1-S5
