# Supplementary material for: Temperature response of Rubisco kinetics in Arabidopsis thaliana: thermal breakpoints and implications for reaction mechanisms
Source: J Exp Bot. 2018 Nov 7;70(1):231–42. doi: 10.1093/jxb/ery355 (PMC6305185; doi:10.1093/jxb/ery355)
Supplement: Supplementary Figures S1-S3 Tables S1-S5 [file ery355_suppl_supplementary_figures_s1-s3_tables_s1-s5.pdf]

Title: Temperature response of Rubisco kinetics in *Arabidopsis thaliana*: thermal breakpoints and implications for reaction mechanisms.

Running title: **Rubisco kinetic thermal breakpoints and reaction mechanisms**

Authors: Ryan A. Boyd, Amanda P. Cavanagh, David S. Kubien, Asaph B. Cousins

Supplementary Figures and Tables

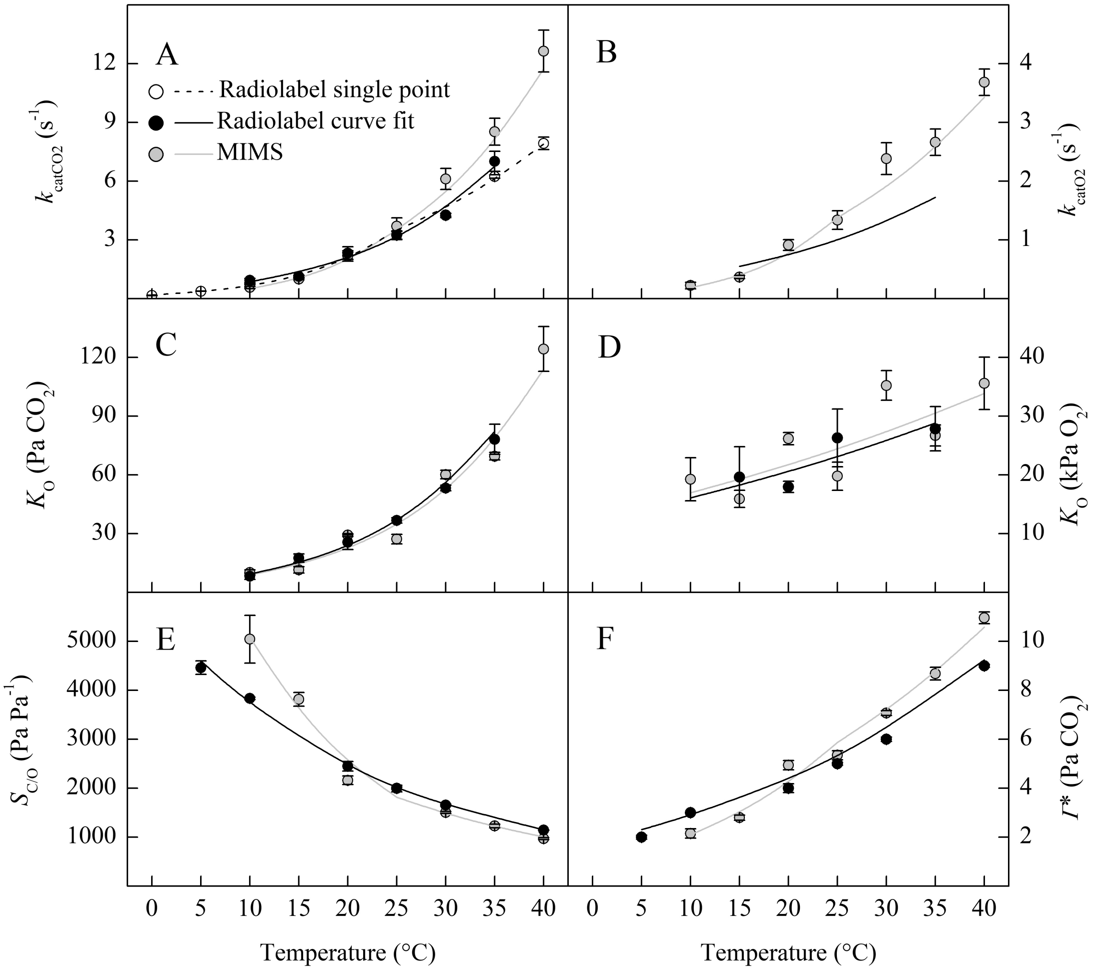

Supplementary Figure 1. Temperature response of Rubisco parameters from *Arabidopsis thaliana* measured using radiolabel and MIMS methods compared to previously published *in vivo* method (Walker et al, 2013). The temperature response of catalytic turnover for CO_2 (k_{catCO2}, Panel A), and O_2 (k_{catO2}, Panel B), the Michaelis constant for CO_2 (K_C, Panel C), and O_2 (K_O, Panel D), the specificity for CO_2 over O_2 ($S_{C/O}$, Panel E), and the CO_2 compensation point in the absence of dark type respiration (Γ*, Panel F) are shown.

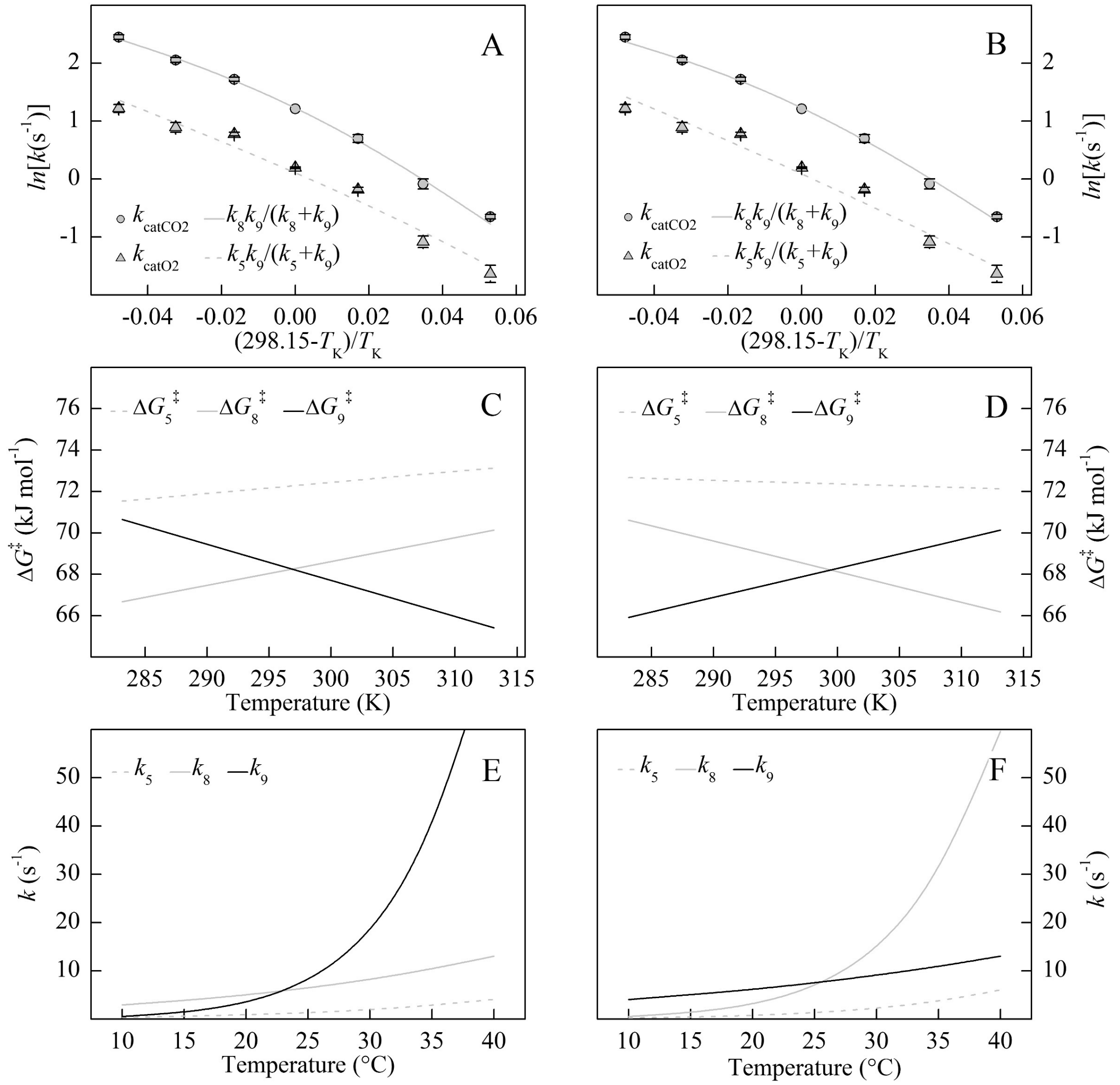

Supplementary Figure 2. Two possible crossover models that result in breakpoints for k_{catCO2} for MIMS data. Each column represents a possible model, for model assumptions see Methods section. The first row shows Arrhenius plots of k_{catCO2} (filled grey circles) and k_{catO2} (filled grey triangles) normalized to zero at 25 °C with model fits k_{catCO2} (solid grey line) and k_{catO2} (dashed grey line). The second row shows the predicted changes in $\Delta G^{\ddagger}$ needed for cleavage of the

oxygenated intermediated (step 5, dashed grey line), carboxylated intermediate (step 8, solid grey line), and RuBP enolization (step 9, solid black line). The third row shows the resulting temperature response of the elementary rate constants for steps 5 (dashed grey line), 8 (solid grey line), and 9 (solid black line); this includes the crossover of rates k_8 and k_9 suggested by Badger and Collatz (1977) to cause an observed breakpoint in k_{catCO2}. Note that given the model assumptions, it is arbitrary if k_8 or k_9 is larger at high temperatures therefor both are shown. While this modeling approach results in breakpoints in k_{catCO2}, Arrhenius plots of k_{catO2} remain essentially linear within the measured temperature range (Panel A and B).

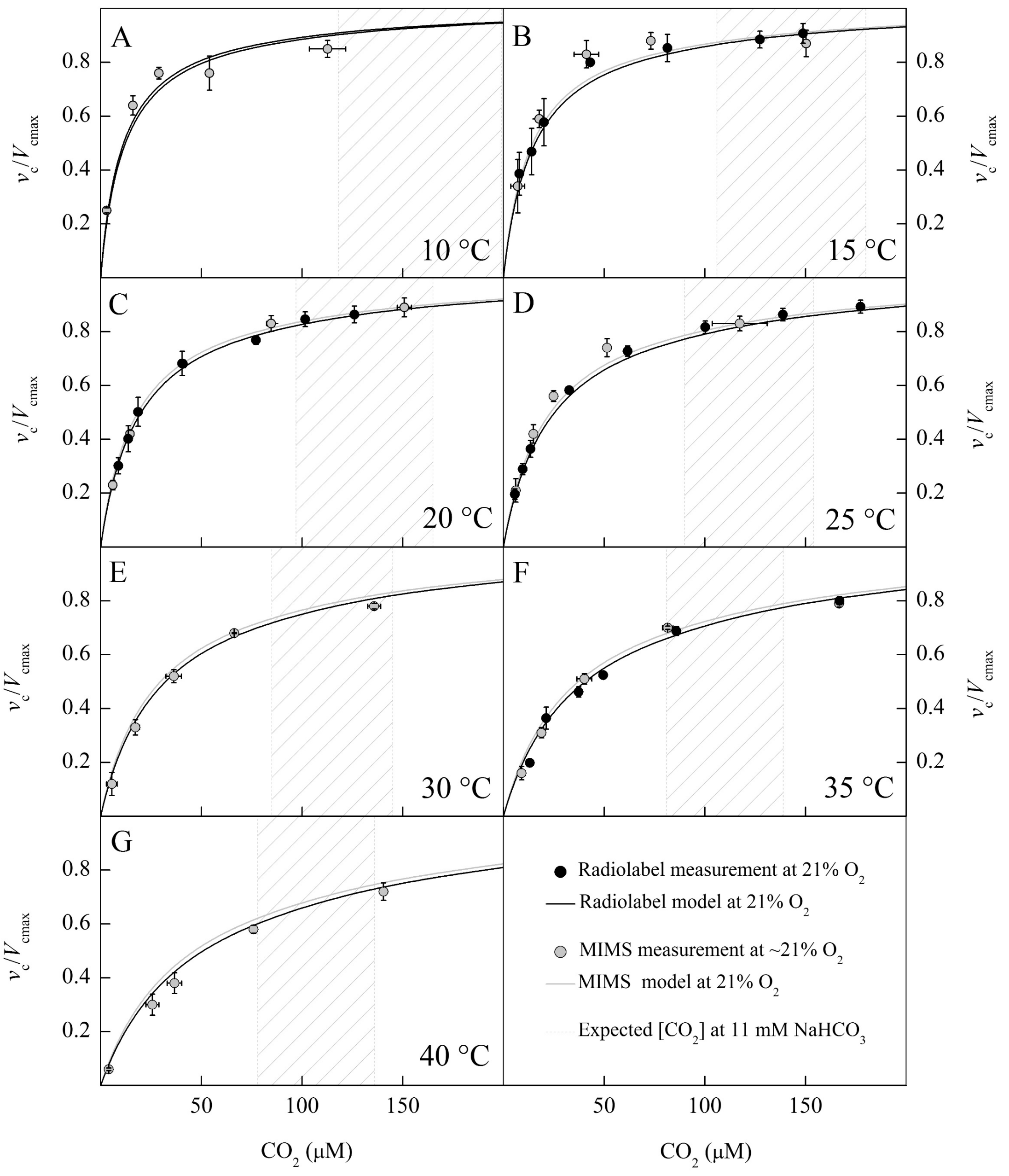

Supplementary Figure 3. CO_2 response curves from 10 to 40 ^{o}C showing measured values from the Radiolabel and MIMS curve fitting methods (filled circles), the predicted response from the modeled K_C and K_O values reported in Table 2 using the Michaelis-Menten equation (solid lines), and the predicted CO_2 concentration used in the Radiolabel single point estimate of V_{cmax}

using 11 mM $NaHCO_3$ (shaded grey box). Because Björkman and Pearcy (1970) suggested that determining the temperature response of k_{catCO2} from a single bicarbonate concentration was inhibiting at low temperatures and sub-saturating at high temperatures, the expected CO_2 concentration given 11 mM $NaHCO_3$ used in the Radiolabel single point k_{catCO2} temperature response was calculated (shaded grey boxes). The CO_2 concentration was calculated from the Henderson-Hasselbalch equation $pH = pK_a + log_{10}(HCO_3^-/CO_2)$ where pH was 8.2 at all temperatures and the temperature response of pK_a from Harned and Bonner (1945) was used for pK_a determined in both 0 and 0.1 M NaCl (Table S4). It was assumed that the only carbon species in solution were CO_2 and the bicarbonate ion such that $C_{total} = CO_2 + HCO_3^-$ where C_{total} is the total amount of inorganic carbon and was equal to 11 mM at all temperatures. Because two pK_a values were used, a range of CO_2 concentrations were calculated at each temperature as indicated by the shaded boxes. The measured values are those measured at approximately 21% O_2 similar to the conditions utilized for the Radiolabel single point estimate of V_{cmax}. The error bars around the measured values indicate standard error for three biological replicates. In order to compare between measurements, each measured value was divided by the modeled V_{cmax} so that the y-axis ranges from 0 to 1, representing v_c/V_{cmax}. To model the predicted v_c/V_{cmax} response to CO_2, Henry's Law was used to convert 21% O_2 and the temperature models of K_C and K_O reported in pressure from Table 2 to units of concentration, these temperature dependent values were used in the Michaelis-Menten model to calculate v_c/V_{cmax} for the range of CO_2 concentrations shown. As shown in the figure, when temperature increases 11 mM $NaHCO_3$ provides less CO_2 indicated by the shaded box moving to lower CO_2 concentrations.

Supplementary Table 1. pKa values used in calculations. The second and third columns are pKa values from Harned and Bonner (1945) used to calculate the shaded area in Supplemental Figure 2. The fourth column is the pKa value from Edsell and Wyman (1958) used to calculate CO_2 concentrations for the Radiolabel assay. The fifth column is the measured pKa value determined by MIMS and used in the calculation of CO_2 concentration for the MIMS assay, where the value is the mean of at least three measurements with standard error shown.

Temperature (°C)	0 M NaCl	0.1 M NaCl	Estimated for Radiolabel	Measured for MIMS	
10	6.46	6.23	6.36	6.22	±0.00
15	6.42	6.19	6.30	6.29	±0.00
20	6.38	6.15	6.25	6.29	±0.01
25	6.35	6.12	6.23	6.22	±0.02
30	6.33	6.09	6.21	6.24	±0.00
35	6.31	6.07	6.19	6.32	±0.00
40	6.30	6.05	6.19	6.25	±0.00

Supplementary Table 2. Average Rubisco kinetic parameters measured at each temperature with ± standard error. The same values are presented in Supplemental Figure 1.

	Radiolabel (curve fit)	Radiolabel (single point)	MIMS
Temperature (°C)		k_{catCO2} (s^{-1})	
0		0.2 ±0.0	
5		0.4 ±0.0	
10	0.9 ±0.1	0.7 ±0.0	0.6 ±0.1
15	1.1 ±0.0	1.1 ±0.1	1.0 ±0.0
20	2.3 ±0.3	2.2 ±0.3	2.2 ±0.2
25	3.2 ±0.2	3.2 ±0.2	3.7 ±0.4
30	4.3 ±0.1	4.3 ±0.1	6.1 ±0.5
35	7.0 ±0.5	6.2 ±0.1	8.5 ±0.7
40		7.9 ±0.3	12.6 ±1.1
Temperature (°C)		k_{catO2} (s^{-1})	
10			0.2 ±0.1
15			0.4 ±0.0
20			0.9 ±0.1
25			1.3 ±0.2
30			2.4 ±0.3
35			2.7 ±0.2
40			3.7 ±0.2
Temperature (°C)		K_C (Pa)	
10	8.2 ±1.6		10.2 ±1.3
15	17.5 ±2.2		11.5 ±1.6
20	25.6 ±3.7		29.2 ±0.7
25	36.7 ±1.4		27.2 ±2.4
30	53.3 ±1.4		60.2 ±2.2
35	78.2 ±7.7		69.5 ±1.9
40			124.3 ±11.4
Temperature (°C)		K_O (kPa)	
10			19.3 ±3.7
15	19.6 ±5.2		15.9 ±1.5
20	18.0 ±1.0		26.2 ±1.0
25	26.3 ±4.9		19.8 ±2.4
30			35.2 ±2.5
35	27.9 ±3.8		26.7 ±1.8
40			35.6 ±4.5
Temperature (°C)		$S_{C/O}$ (Pa Pa^{-1})	
5	4461 ±137		
10	3835 ±24		5045 ±487
15			3816 ±138
20	2448 ±96		2161 ±87
25	2001 ±23		1994 ±67
30	1655 ±22		1505 ±21
35			1228 ±36
40	1144 ±8		971 ±21

Supplementary Table 3. The $\Delta H^{\ddagger}$ and $\Delta S^{\ddagger}$ calculated for the $\Delta G^{\ddagger}$ values presented in Figure 5 using Equation 18 from the main text.

Method	Parameter	Temperature (°C)	Value		
Radiolabel	$\Delta H_3^{\ddagger} - \Delta H_6^{\ddagger}$	5 - 40	28.69	±0.52	kJ mol^{-1}
	$\Delta S_3^{\ddagger} - \Delta S_6^{\ddagger}$	5 - 40	33.04	±1.67	J mol^{-1} K^{-1}
MIMS	$\Delta H_3^{\ddagger} - \Delta H_6^{\ddagger}$	10 - 25	46.67	±4.20	kJ mol^{-1}
		25 - 40	36.61	±0.21	kJ mol^{-1}
	$\Delta S_3^{\ddagger} - \Delta S_6^{\ddagger}$	10 - 25	94.05	±14.33	J mol^{-1} K^{-1}
		25 - 40	59.75	±0.73	J mol^{-1} K^{-1}

Supplementary Table 4. The $\Delta H^{‡}$ and $\Delta S^{‡}$ calculated for the $\Delta G^{‡}$ values presented in Figure 6 using Equations 16 and 17.

**Method**	**Parameter**	**Temperature (°C)**	**value**		
Radiolabel	$\Delta H_{kcatCO2}{}^{‡}$	10 - 35	57.21	±3.97	kJ mol^{-1}
	$\Delta S_{kcatCO2}{}^{‡}$	10 - 35	-43.63	±13.29	J mol^{-1} K^{-1}
MIMS	$\Delta H_{kcatO2}{}^{‡}$	10 - 25	87.31	±6.70	kJ mol^{-1}
		25 - 40	46.88	±4.20	kJ mol^{-1}
	$\Delta S_{kcatO2}{}^{‡}$	10 - 25	50.85	±22.34	J mol^{-1} K^{-1}
		25 - 40	-84.61	±42.84	J mol^{-1} K^{-1}
	$\Delta H_{kcatCO2}{}^{‡}$	10 - 25	86.93	±1.79	kJ mol^{-1}
		25 - 40	60.22	±2.05	kJ mol^{-1}
	$\Delta S_{kcatCO2}{}^{‡}$	10 - 25	57.61	±6.75	J mol^{-1} K^{-1}
		25 - 40	-31.90	±6.00	J mol^{-1} K^{-1}

Supplementary Table 5. The Δ$H^‡$ and Δ$S^‡$ calculated for the Δ$G^‡$ values presented in Figure 7 using Equations 9 through 15 from the main text.

**Method**	**Temperature (°C)**	**Step**	**Δ$H^‡$ (kJ mol^{-1})**		**Δ$S^‡$ (J mol^{-1} K^{-1})**	
Radiolabel	10 - 35	3	24.2	±3.9	-154.6	±13.0
		5	37.8	±8.3	-116.9	±26.2
		6	-5.9	±3.9	-244.2	±13.0
		8	--	--	-38.7	±13.2
		9	57.2	±3.9	-37.2	±13.2
		10	--	--	-30.2	±13.2
MIMS	10 - 25	3	77.0	±38.8	25.4	±13.4
		5	92.0	±7.7	67.5	±26.1
		6	33.0	±3.3	-111.4	±10.8
		8	101.5	±2.9	110.8	±9.5
		9	61.0	±1.5	-23.5	±5.3
		10	--	--	-16.5	±5.3
	25 - 40	3	23.2	±6.0	-155.2	±19.6
		5	45.1	±3.1	-89.9	±10.5
		6	-11.6	±6.1	-261.0	±20.0
		8	--	--	-25.1	±5.3
		9	61.0	±1.5	-23.5	±5.3
		10	--	--	-16.5	±5.3
